# Cost awareness of radiological studies among doctors at Universitas Academic Hospital in Bloemfontein, South Africa

**DOI:** 10.4102/sajr.v25i1.2188

**Published:** 2021-09-20

**Authors:** Khanyisa N. Mrwetyana, Jacques Janse van Rensburg, Gina Joubert

**Affiliations:** 1 Department of Clinical Imaging Sciences, Faculty of Health Sciences, University of the Free State, Bloemfontein, South Africa; 2 Department of Biostatistics, Faculty of Health Sciences, University of the Free State, Bloemfontein, South Africa

**Keywords:** cost awareness, radiological studies, imaging, estimation, accuracy

## Abstract

**Background:**

South Africa has high healthcare expenses. Improving cost-consciousness could decrease government expenditure on healthcare.

**Objectives:**

To determine cost awareness of radiological studies among doctors at a tertiary hospital. The objective was met by assessing the accuracy of cost estimation according to the level of training and speciality, whether participants had received prior education/training related to cost awareness and if they had a desire to learn more about the cost of radiological imaging.

**Method:**

A cross-sectional survey was conducted in six clinical departments at Universitas Academic Hospital using an anonymous questionnaire that determined doctors’ cost awareness of five radiological studies. Each radiological study was answered using six different cost ranges, with one correct option. Costs were based on the Department of Health’s 2019 Uniform Patients Fee Schedule (UPFS).

**Results:**

In total, 131 (67.2%) of 195 questionnaires distributed to registrars and consultants were returned. Overall, low accuracy of cost estimation was observed, with 45.2% of the participants choosing only incorrect options. No participant estimated all five costs correctly. Only the Internal Medicine clinicians demonstrated a significant difference between registrars and consultants for the number of correct answers (median 0 and 1, respectively) (*p* = 0.04). No significant differences were found between specialities stratified by registrars/consultants. Most participants (88.6%) would like to learn about imaging costs. Only 2.3% of the participants had received prior education/training related to cost awareness of radiological studies.

**Conclusion:**

Doctors were consistently inaccurate in estimating the cost of radiological studies. Educating doctors about the cost of radiological imaging could have a positive effect on healthcare expenditure.

## Introduction

It is estimated that patient-care decisions made by doctors are responsible for more than 80% of the healthcare expenditure.^1^ Doctors are responsible for the radiological studies requested and can act as gatekeepers to ensure that resources are used judiciously and efficiently. South Africa, a developing country, has limited resources and a high burden of disease. With the current economic situation, the South African government will benefit from mitigating fruitless and wasteful expenditure on healthcare costs. For this to be realised, it has to start with incorporating cost-consciousness into medical practice.^2^

When doctors are aware of the costs of radiological studies, it may reduce the number of studies requested, which would translate into reduced healthcare costs.^3,4^ Decreasing unnecessary costs would mean that funds could be saved and reallocated to other critical healthcare burdens in South Africa such as tuberculosis, HIV and AIDS.^5^ Inappropriate use of radiological studies adds to healthcare costs without improving the quality of care provided to patients. According to the South African Competition Commission’s Health Market Inquiry, claims submitted to medical schemes for radiological studies increased by approximately 11% per year between 2011 and 2014.^6^

International studies report that the number of radiological studies requested has increased drastically in the past 20 years. Medical imaging utilisation has grown faster than any other medical service, which puts a massive strain on the healthcare expenditure, and can result in the unsustainability of the healthcare system. The increase in medical imaging is partially due to the use of new imaging techniques but could mostly be attributed to overutilisation of radiological studies. Overutilisation of radiological studies adds to unnecessary expenditure and contributes to unwarranted costs to the healthcare system.^7,8,9,10^ It also exposes patients to unnecessary radiation, which in turn predisposes patients to increased cancer risk.

It has been reported that approximately 33% of healthcare spending is duplicative, pointless and avoidable. They may also aggravate the patient’s clinical condition.^9^ Yet, the majority of doctors are unaware of the costs of radiological studies they request. Several studies have also shown that doctors inaccurately estimate the cost of radiological studies they request and are unlikely to consider the effects of over-investigation on patients and the healthcare system.^2,11,12,13^ In Saudi Arabia, only 3.4% of surgeons were cognizant of the costs of imaging investigations.^2^ A Canadian study^13^ found that emergency physicians had limited awareness of the costs of pharmaceutical, laboratory and radiological studies. These physicians overestimated pharmaceutical agents and laboratory costs, opposed to frequently underestimating the costs of radiological studies. A disregard for costs related to radiological imaging was attributed to a lack of health economics teaching in medical schools and insufficient training on costs during residency.^13^

Steyn and Gebremariam,^14^ in a recent study, reported on the cost of violence-related medical imaging in a trauma unit of a tertiary hospital in the Free State Province. However, to the best of the authors’ knowledge, no studies specifically focussing on radiological cost awareness have been published in South Africa.

This study aimed to determine cost awareness of radiological studies among doctors at Universitas Academic Hospital, Bloemfontein, South Africa. The objectives were to assess (1) whether there were differences in the accuracy of cost estimations according to different levels of training and speciality and (2) whether the participants had received any prior education or training related to cost awareness of radiological studies, and if they desired to learn about the cost of radiological imaging.

## Methods

### Study design

A cross-sectional observational study was conducted to assess the cost awareness of radiological studies among registrars and consultants at Universitas Academic Hospital, Bloemfontein, South Africa.

### Research setting and sampling method

The study was conducted among medical professionals employed in six clinical departments at Universitas Academic Hospital, Bloemfontein, South Africa. The participants were recruited from the departments of Clinical Imaging Sciences, Internal Medicine, Paediatrics and Child Health, General Surgery, Oncology and Obstetrics and Gynaecology.

### Data collection

A questionnaire, which was in English, was developed to collect the data. The questionnaire consisted of three sections: (1) demographic information including age, sex, speciality, level of training, involvement in private practice; (2) estimation of radiological study cost and (3) the participant’s desire to learn about imaging costs and any prior education or training related to cost awareness of radiological studies.

The Uniform Patients Fee Schedule (UPFS) is a fee schedule used to bill externally funded patients using public hospitals across South Africa. Imaging studies on the UPFS are categorised from category A to E according to the complexity of the investigation, with category A procedures characterised as least expensive and category E most expensive. Each category has two prices: a facility fee (depending on the level of the hospital, which may be level 1, 2 or 3 as determined by the extent of services rendered by the specific hospital) and a professional fee (depending on the level of training of the healthcare professional who performs or interprets the radiological study).^14,15^ The 2019 UPFS radiological fees are presented in Table 1.^15^

**TABLE 1:** South African Department of Health 2019 Uniform Patient Fee Schedule: Tariffs for radiological studies according to hospital level.^15^

Category of investigation	Professional fee	Facility fee		
		Level 1†	Level 2‡	Level 3§
**Category A – facility fee**		R80.00	R80.00	R89.00
Allied health practitioner	R77.00	R157.00	R157.00	R166.00
General medical practitioner	R78.00	R158.00	R158.00	R167.00
Specialist medical practitioner	R146.00	R226.00	R226.00	**R235.00**
**Category B – facility fee**		R219.00	R219.00	R250.00
Allied health practitioner	R205.00	R424.00	R424.00	R455.00
General medical practitioner	R210.00	R429.00	R429.00	R460.00
Specialist medical practitioner	R409.00	R628.00	R628.00	**R659.00**
**Category C – facility fee**		R507.00	R507.00	R579.00
General medical practitioner	R326.00	R833.00	R833.00	R905.00
Specialist medical practitioner	R1000.00	R1507.00	R1507.00	**R1579.00**
**Category D – facility fee**		R1013.00	R1013.00	R1156.00
General medical practitioner	R649.00	R1662.00	R1662.00	R2169.00
Specialist medical practitioner	R1997.00	R3010.00	R3010.00	**R3153.00**
**Category E – facility fee**		R2582.00	R2582.00	R2952.00
General medical practitioner	R2391.00	R4973.00	R4973.00	R5343.00
Specialist medical practitioner	R4985.00	R7567.00	R7567.00	**R7937.00**

Source: Adapted from Department of Health, Province of the Western Cape. Provincial Gazette Extraordinary No. 8069 [homepage on the Internet]. Uniform Patient Fee Schedule regulations for health care services rendered by the Western Cape Department of Health; 2019 [cited 2021 Apr 10]. Available from: https://www.google.co.za/url?sa=t&rct=j&q=&esrc=s&source=web&cd=&cad=rja&uact=8&ved=2ahUKEwjjoa_hwvPvAhVcQ0EAHcOsCHMQFjAAegQIBRAD&url=https%3A%2F%2Farchive.opengazettes.org.za%2Farchive%2FZA-WC%2F2019%2Fprovincialgazette-ZA-WC-no-8069-dated-2019-03-29.pdf&usg=AOvVaw1v6ixy2rCcg4jPldLX7rQj

Note: Prices shown in bold text applied to Universitas Academic Hospital at the time of the study, plus R105.00 added for contrast medium in all studies requiring contrast.

All fees mentioned in this Table are in South African Rand.

† , **Level 1** hospital: where limited specialist or no specialist services are rendered, but basic diagnostic and therapeutic services are available

‡ , **Level 2** hospital: has at least two of the following specialist services: General Surgery, Orthopaedic Surgery, Internal Medicine, Paediatrics, and Gynaecology and Obstetrics

§ , **Level 3** hospital: where all specialist services are continuously rendered, or those specialist services are rendered as determined by the Head of Department for the DOH.^15^

Concerning the estimation of the cost of radiological studies, five different imaging modalities were included: (1) two-view chest X-ray (posteroanterior and lateral projections), (2) non-contrasted CT of the brain, (3) MRI of the brain without contrast, (4) contrast-enhanced CT of the abdomen and pelvis and (5) abdominal ultrasound. These modalities were chosen because they were among the most frequently requested radiological studies. Doctors were provided with six different cost ranges to choose from for each modality, with only one correct option. The costs were based on the South African National Department of Health’s 2019 UPFS.^15^

**FIGURE 1:**
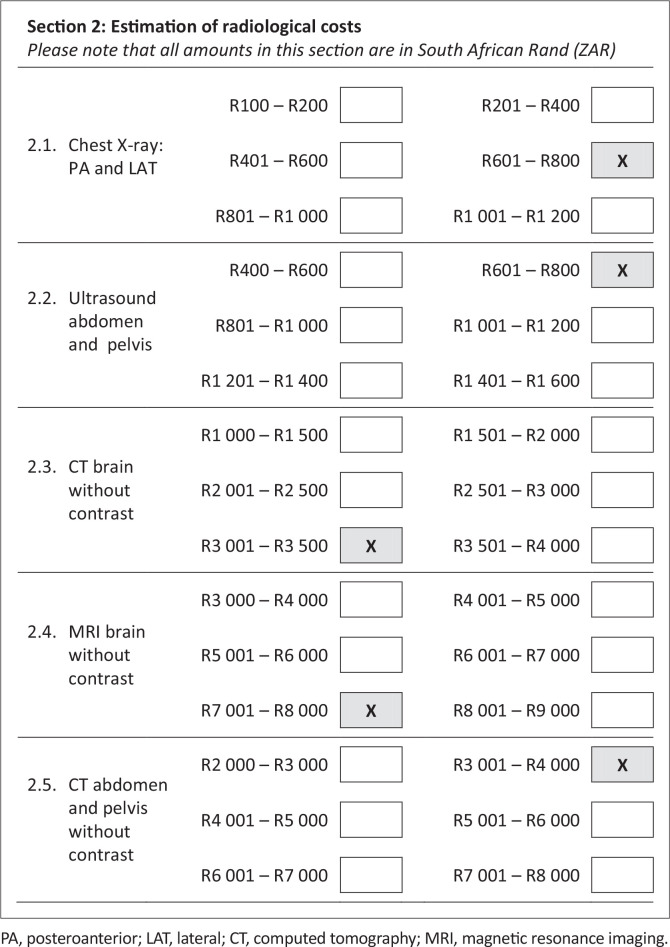
Cost estimation component of the questionnaire with radiological costs from which the participants had to select one correct option (the correct options are indicated by X).

Figure 1 represents the section of the questionnaire pertaining to cost estimation of radiological studies. An open-ended question on why they would want to learn about the cost of radiological studies was also included in the questionnaire.

In total, 195 doctors qualified to participate in this study. The questionnaires were distributed and collected by the principal researcher at the academic meetings of the various departments. Participation was voluntary. The questionnaires were completed immediately and anonymously, after which the completed forms were placed in a sealed box.

### Data analysis

The principal researcher entered the data into a Microsoft Excel spread sheet designed for the purpose of the study. Analysis was done by the Department of Biostatistics, Faculty of Health Sciences, University of Free State. In cases where a cost estimation question was not answered, it was assumed that the respondent did not know the correct answer. Categorical variables were summarised by frequencies and percentages and numerical variables by medians and percentiles. Subgroups were compared using chi-square or Fisher’s exact tests (categorical variables) and median tests (numerical variables).

### Ethical considerations

Ethical approval was obtained from the Health Sciences Research Ethics Committee (HSREC) of the University of the Free State (ethics clearance number: UFS-HSD2018/1588/2304). Permission to perform the study was obtained from the appropriate University of Free State authorities and the heads of departments involved in this study. All questionnaires were anonymous and completion of the questionnaire implied consent.

## Results

Of the 195 questionnaires distributed, 131 (67.2%) were completed and returned. As shown in Table 2, approximately 60% of the participants were male (*n* = 74/125; 59.2%; missing data: *n* = 6), and approximately half were between 26 and 35 years of age (*n* = 64; 48.9%). Most respondents were from the Departments of Paediatrics and Child Health and Internal Medicine, with both at 22.9% (*n* = 30), followed by General Surgery (*n* = 27; 20.6%). Participants from Clinical Imaging Sciences represented only 12.2% (*n* = 16) of the study sample.

**TABLE 2:** Demographic information of participating doctors.

Variable	*n*	%
**Gender (*n* = 125)**		
Male	74	59.2
Female	51	40.8
**Age group (*n* = 131)**		
≤ 25 years	2	1.5
26–35 years	64	48.9
36–45 years	40	30.5
46–55 years	17	13
56–60 years	2	1.5
> 60 years	6	4.6
**Clinical department (*n* = 131)**		
Clinical Imaging Sciences	16	12.2
General Surgery	27	20.6
Internal Medicine	30	22.9
Paediatrics and Child Health	30	22.9
Obstetrics and Gynaecology	17	13
Oncology	11	8.4
**Position (*n* = 131)**		
Registrar	89	67.9
Consultant	42	32.1

Table 3 summarises the distribution of registrars and consultants per department. Proportionally, the Department of Clinical Imaging Sciences had the most registrars among the total participants (*n* = 15/16; 93.8%) compared to consultants, while Internal Medicine had 30 participants of whom 16 (53.3%) were registrars.

**TABLE 3:** Position of respondents per department.

Department	Registrars		Consultants	
	*n*	%	*n*	%
Clinical Imaging Sciences (*n* = 16)	15	93.8	1	6.3
General Surgery (*n* = 27)	20	74.1	7	25.9
Internal Medicine (*n* = 30)	16	53.3	14	46.7
Paediatrics (*n* = 30)	17	56.7	13	43.3
Obstetrics and Gynaecology (*n* = 17)	14	82.4	3	17.7
Oncology (*n* = 11)	7	63.6	4	36.4

**Total (*n* = 131)**	**89**	**67.9**	**42**	**32.1**

Most of the participating doctors were registrars (*n* = 89; 67.9%), with 25 (28.1%) being in their fourth year of training. Approximately one-third (*n* = 42; 32.1%) of the study sample were consultants, of whom 39% (*n* = 16) were also involved in private practice, in addition to their public sector commitments. The Department of Clinical Imaging Sciences had three consultants of which only one (33.3%) participated in the study. Out of 25 consultants in Internal Medicine, 14 (56.0%) participated, while 13 (59.1%) of the 22 consultants in Paediatrics and Child Health department participated in the study. The response rate of consultants in Obstetrics and Gynaecology, Surgery and Oncology was 30.0% (3/10), 70.0% (7/10) and 100.0% (4/4), respectively. There was a 100% (15/15) response rate of Clinical Imaging Sciences registrars, while Paediatrics and Child Health had the lowest response rate of registrars (*n* = 17/30; 56.7%). Surgery, Obstetrics and Gynaecology, Oncology and Internal Medicine registrars’ response rate was 83.3%, 82.4%, 77.8% and 61.5%, respectively.

An overall low level of accuracy in cost estimation was observed, with 45.0% (*n* = 59) of the participants estimating none of the costs correctly. None of the participants made more than three correct estimations. A total of 49 (37.4%) participants estimated only one radiological study cost correctly, with only 3.1% estimating three costs correctly. The median number of correct responses was one, with an interquartile range of 0–1. The only significant difference between registrars and consultants on the number of correct estimations (median 0 and 1, respectively; *p* = 0.04), was observed among the Department of Internal Medicine participants. No significant differences were found between specialities stratified by registrars/consultants.

Table 4 summarises the results of the cost estimation for the five different radiological studies. The two-view chest radiography was the study with the highest number of participants underestimating the cost (*n* = 99; 75.6%), while the cost of abdominal and pelvic CT with contrast was overestimated by 84.7% (*n* = 111) of the participants. The cost of non-contrasted brain CT was estimated correctly by 32 (24.4%) participants, while only 7 (5.3%) participants gave a correct estimation of the cost of abdominal/pelvic CT with contrast.

**TABLE 4:** Accuracy of cost estimation of radiological studies by participating doctors (*n* = 131).

Radiological study (price category)	Correct estimation		No answer provided		Under-estimation		Over-estimation	
	*n*	%	*n*	%	*n*	%	*n*	%
Chest X-ray: PA and LAT (B)	18	13.7	1	0.8	99	75.6	13	9.9
Abdomen and pelvic U/S (B)	16	12.2	2	1.5	23	17.6	90	68.7
Non-contrasted CT brain (D)	32	24.4	1	0.8	61	46.6	37	28.2
Non-contrasted MRI brain (E)	26	19.8	1	0.8	68	51.9	36	27.5
CT abdomen and pelvis with contrast (D)	7	5.3	2	1.5	11	8.4	111	84.7

PA, posteroanterior; LAT, lateral; U/S, ultrasound; CT, computed tomography; MRI, magnetic resonance imaging.

The majority of participants (*n* = 116; 88.5%) indicated an interest in obtaining more information on the cost of radiological studies. Twenty-three (17.2%) participants did not provide a reason for wanting to learn more about the cost of radiological studies, and 38 (29.2%) of those who did answer the question indicated that they wanted to manage resources effectively and be more cost-effective and better gatekeepers. Only 6.3% of the participants indicated that they wanted to be better advisors for the patients and the clinicians. Of those participants who would like to learn about the cost of radiological studies, 7.3% said that they wanted to be cost-conscious or cost-aware, 3.2% indicated that it would improve patient care and clinical judgement.

There were 2.1% participants who wanted to learn about the cost of radiological studies because this information would be beneficial for them when they work in private practice. Of the 15 participants who indicated that they did not have an interest in learning about the cost of radiological studies, 4 (26.7%) indicated that cost was irrelevant when a radiological study is indicated. Only 3 (2.3%) of the participants had received prior education or training related to cost awareness of radiological studies.

## Discussion

The results of this study were similar to international studies conducted on cost awareness of radiological studies and a Johannesburg-based South African study done on the cost awareness of medical consumables among healthcare professionals.^2,11,12,13^ These studies have shown that healthcare professionals are unaware of the costs.^2,11,12,13^ Vijayasarathi et al.^11^ showed that radiology trainees had poor knowledge of the cost of radiological studies, with 45.1% of the participants not estimating any of the costs correctly, which was comparable with the findings from our study. In their study, only 0.3% of the participants estimated all five examinations correctly,^11^ which was negligibly better than 0.0% of the participants in our study.

Poor knowledge of costs could probably be attributed to the lack of cost awareness education in medical schools and postgraduate registrar training programmes. In our study, two of the three participants who indicated that they had received prior education or training related to cost awareness of radiological studies, were consultants. Physicians should be the gatekeepers of healthcare expenditure and should play a critical role in the use of healthcare resources. They have an ethical obligation to render high-value, high-quality healthcare and limit unnecessary costs that do not improve a patient’s clinical outcome.^16^

Healthcare professionals should be informed regarding not only the benefits or effectiveness of diagnostic investigations and drugs but also about their costs. When requesting a radiological investigation, cost should be taken into consideration. When cost consciousness is incorporated in the medical school curriculum, physicians will better understand the need for financial resource management, consequently curbing unnecessary and improper diagnostic investigations and therapies that do not improve patient care, but rather add to healthcare expenditure. Medical school programmes should also expose their students and registrars to knowledge of healthcare management, health service delivery and how medical care is financed to increase their consciousness of the healthcare systems they work in, thereby empowering them to make informed decisions.^16^

When doctors are aware of the costs of radiological studies, it may lead to a more judicious use of radiological studies, reducing the number of unnecessary investigations, which translates into a reduction in the healthcare expenditure.^3,4^ An educational intervention study on abdominal imaging performed by Covington et al.^4^ incorporated the American College of Radiology appropriateness criteria, lectures on general principles of cost-conscious medicine and discussions of actual hospital costs for commonly ordered abdominal investigations. They compared the number of abdominal investigations requested before and after the study and found a statistically significant reduction in the average abdominal CT scans ordered per patient. They also reported a substantial cut in expenditure of more than $80 000.00.^4^ Kruger et al.^17^ conducted a pre- and post-interventional study where they displayed radiation exposure and cost of diagnostic imaging on the electronic order form. They reported a decrease in the number of CT scans and MRIs ordered compared to ultrasound, after the radiation exposure and costs related to the investigations were known. Most of the surveyed clinicians wanted the displays to continue, as it influenced their ordering behaviour, though most of them admitted that the radiation exposure influenced their decision more than the cost.^17^ These two studies^4,17^ prove that cost-consciousness among healthcare professionals does reduce costs.

The increase in radiological studies usage is partly due to the availability of more advanced and high-tech imaging modalities, but overutilisation also plays a role^7,18^ and contributes to excessive expenditure and unjustifiable costs to the healthcare system. Among many reasons for overutilisation, uncertainty or a lack of knowledge among requesting doctors about imaging indications and costs that result in the inappropriate use of imaging studies plays an important role.^18,19,20^

Several clinical imaging guidelines are available that clinicians can use to justify the performance of a particular radiological study. Justification in radiology refers to the appropriate application of radiologic imaging modalities.^20^ We do not have guidelines specifically applicable to the South African setting. However, guidelines that have been developed in the United Kingdom and the United States of America are the Royal College of Radiologists guidelines^21^ and the American College of Radiology appropriateness criteria,^22^ respectively. Familiarising themselves with one of these clinical imaging guidelines appropriate for different medical or surgical conditions will assist clinicians in choosing the imaging study most suitable for a particular medical condition. Individual hospitals or the Radiological Society of South Africa (RSSA) can decide which of these guidelines can be followed in South African hospitals to ensure uniformity. These are evidence-based guidelines that have been developed to assist primary physicians in making the most appropriate decisions about imaging and patient management. Applying these guidelines will assist clinicians to improve the quality of care, guarantee advantageous use of radiological investigations and reduce healthcare expenditure.^10,23^

A report by the American Health Insurance Plans alleged that up to 50.0% of all high-tech imaging is unnecessary because it does not provide beneficial information.^8^ A retrospective international study^24^ analysed outpatient CT and MRI appropriateness based on the American College of Radiology Appropriateness Criteria. It was found that 26.0% of the studies were inappropriate.^24^ In contrast, a South African study conducted in the Western Cape Province showed that 6.4% of scans were inappropriate,^10^ which was remarkably less than the findings of the American study.^24^

## Limitations

The objectives of the study were to assess whether differences occurred in the accuracy of cost estimations according to the level of training, involvement in private practice and the number of years in practice. However, the numbers were too small to investigate these issues and the number of participants representing the respective specialities differed vastly.

## Conclusion

Doctors were consistently inaccurate in estimating the cost of the radiological studies. As doctors are largely responsible for healthcare expenditure, the results of this research suggest that educating doctors about the cost of radiological imaging can positively affect healthcare expenditure.

It is encouraging that the majority of doctors indicated an interest in learning about the cost of imaging, which would have a positive impact on resource management. Previous studies have proven that incorporating cost-consciousness into medical practice does reduce the number of requested imaging, which ultimately translates into a reduction in healthcare costs. Developing or using existing clinical imaging guidelines to justify the performance of a particular radiological study will also contribute to a reduction in wasteful expenditure.
